# Patient Attitudes Towards Ambient Artificial Intelligence in Clinical Consultations

**DOI:** 10.7759/cureus.115822

**Published:** 2026-09-05

**Authors:** Sam Kathiramalai, Theodore Davis, Gavin Schaller

**Affiliations:** 1 Trauma and Orthopaedics, West Hertfordshire Teaching Hospitals National Health Service (NHS) Trust, Watford, GBR; 2 Orthopaedics, Brunel University of London, Watford, GBR; 3 Trauma and Orthopaedics, Watford General Hospital, London, GBR

**Keywords:** ambient artificial intelligence, clinical consultation, consent, healthcare technology, patient attitudes, patient safety

## Abstract

Background

Ambient Artificial Intelligence (AI) technologies are increasingly being integrated into clinical practice to support documentation. These systems employ speech recognition, talk-to-text technology, generative intelligence and large language models (LLMs) to automatically transcribe and summarise clinician-patient consultations into structured clinical notes. While it may reduce documentation burden and enable patient-focused interactions, there remains limited evidence regarding patients' perspectives, experiences and the acceptability of ambient AI use in healthcare settings.

Objective

Our study aims to explore patients' awareness, attitudes, consent preferences and perceived comfort factors regarding the use of ambient AI during consultations.

Methods

A cross-sectional anonymous questionnaire survey was conducted among 55 adult patients attending an orthopaedic fracture clinic. The questionnaire included demographic items, Likert-scale questions (Q9-Q21; scored 1-5) assessing attitudes towards ambient AI use, closed-ended preference questions and open-ended free-text responses. Quantitative data were analysed using descriptive statistics, while qualitative free-text responses were analysed using thematic analysis to identify recurring themes and perspectives.

Findings

General awareness of AI in healthcare was high (72.7%), but detailed knowledge of ambient AI remained limited, with only 10.9% reporting a strong understanding. Participants showed moderate acceptance and trust in clinicians, while expressing concerns about transparency, privacy, consent, openness, accuracy and preservation of patient-clinician relationships during AI use.

Conclusions

As the adoption of AI in healthcare continues to expand, its implementation strategies should prioritise clinician-led explanation and transparency, active and ongoing consent processes, appropriate human oversight and strong data governance safeguards. Recognising variation in patient attitudes, preferences and concerns, accessible and meaningful opt-out mechanisms are essential to maintain patient autonomy, trust and acceptability.

## Introduction

Artificial intelligence (AI) is increasingly being integrated into healthcare, with applications spanning diagnostic support, predictive modelling and analysis, administrative management, and clinical workflow optimisation. One emerging application is ambient AI, which refers to technologies that passively capture and process spoken clinical interactions to generate documentation, summaries, and other workflow outputs. In healthcare settings, these systems are commonly deployed as ambient AI scribes that listen to clinician-patient consultations and automatically generate draft clinical notes for clinician review and approval. By automating documentation tasks, ambient AI aims to reduce the administrative burden associated with electronic health records, improve workflow efficiency and allow clinicians to focus more fully on patient care [1,2]. Products like Heidi Health, Nuance DAX and others are now used in primary care, hospital outpatient departments and specialist settings [3,4].

The rapid adoption of ambient AI has occurred against a backdrop of longstanding concerns regarding the burden of clinical documentation, cognitive overload and clinician burnout. Requirements associated with electronic health records have been identified as a major contributor to administrative workload, often diverting clinician attention away from direct patient care and contributing to after-hours work. Proponents argue that by automating note generation, ambient AI allows clinicians to engage more naturally during consultations, potentially enhancing both patient experience and the quality of clinical encounters [5,6].

Despite these potential benefits, integrating ambient AI into clinical practice raises important ethical, legal and relational considerations. Key concerns include patient privacy, informed consent, confidentiality, data governance, secondary use of recorded information and the accuracy of AI-generated documentation [7-9]. The presence of passive recording technologies within clinical encounters may also influence the therapeutic relationship and patients' perceptions of trust, autonomy and/or control over their personal health information. Understanding patient attitudes towards ambient AI and how acceptance may vary across demographic groups is, therefore, essential to informing responsible implementation and ensuring that adoption aligns with patient expectations and preferences.

Previous research has identified trust in healthcare providers, perceived benefits and risks, control over personal information and the quality of communication about new technologies as important determinants of patient acceptance of healthcare innovations [10]. While these themes are likely to be highly relevant to ambient AI, empirical evidence regarding patient perspectives remains limited, as existing research has largely focused on clinician experiences [4,11] with relatively few studies examining patient attitudes in the clinical populations.

This study aimed to address this gap by investigating patient perspectives on the use of ambient AI during clinical consultations. Specifically, it sought to assess patient awareness of ambient AI, explore attitudes towards its use, examine preferences regarding consent and notification, and identify factors that would increase comfort and trust in its implementation. Using a mixed-methods questionnaire administered to adult patients attending an orthopaedic fracture clinic, this study provides insights that may inform future implementation strategies, clinical governance frameworks, and policy development for the responsible use of ambient AI in healthcare.

## Materials and methods

Study design

An anonymous, non-interventional, mixed-methods cross-sectional questionnaire study was conducted to evaluate patient attitudes towards ambient AI in healthcare consultations. The data was collected from patients prior to attending their outpatient clinic appointment in an orthopaedic fracture clinic in a district general hospital. The study employed a mixed-methods approach, combining quantitative analysis of responses to structured questionnaires and qualitative thematic analysis of free-text comments.

Setting and participants

The sample population included all adults (≥18 years old) who visited the fracture clinic in an orthopaedic outpatient clinic during 2026. Convenience sampling was used; patients were asked to complete the questionnaire in the waiting room, voluntarily. The inclusion criteria were that participants had to be able to read and understand the questionnaire and give a voluntary response. Those with severely impaired cognitive function who were not provided with appropriate support were excluded.

Implied informed consent was obtained through voluntary completion and submission of the questionnaire. Participants were advised that participation was entirely voluntary, that they were free to omit any question and that anonymised responses would be used for analysis and reporting. No direct personal identifiers were collected.

Questionnaire content

The questionnaire (Figure 1) comprised five sections. Section 1 collected non-identifiable demographic information, including age group, gender, educational attainment, frequency of healthcare use and level of engagement with digital technology. Section 2 assessed participants' prior awareness of AI in healthcare and ambient AI, as well as their self-reported understanding of how ambient AI functions in clinical settings.

**Figure 1:**
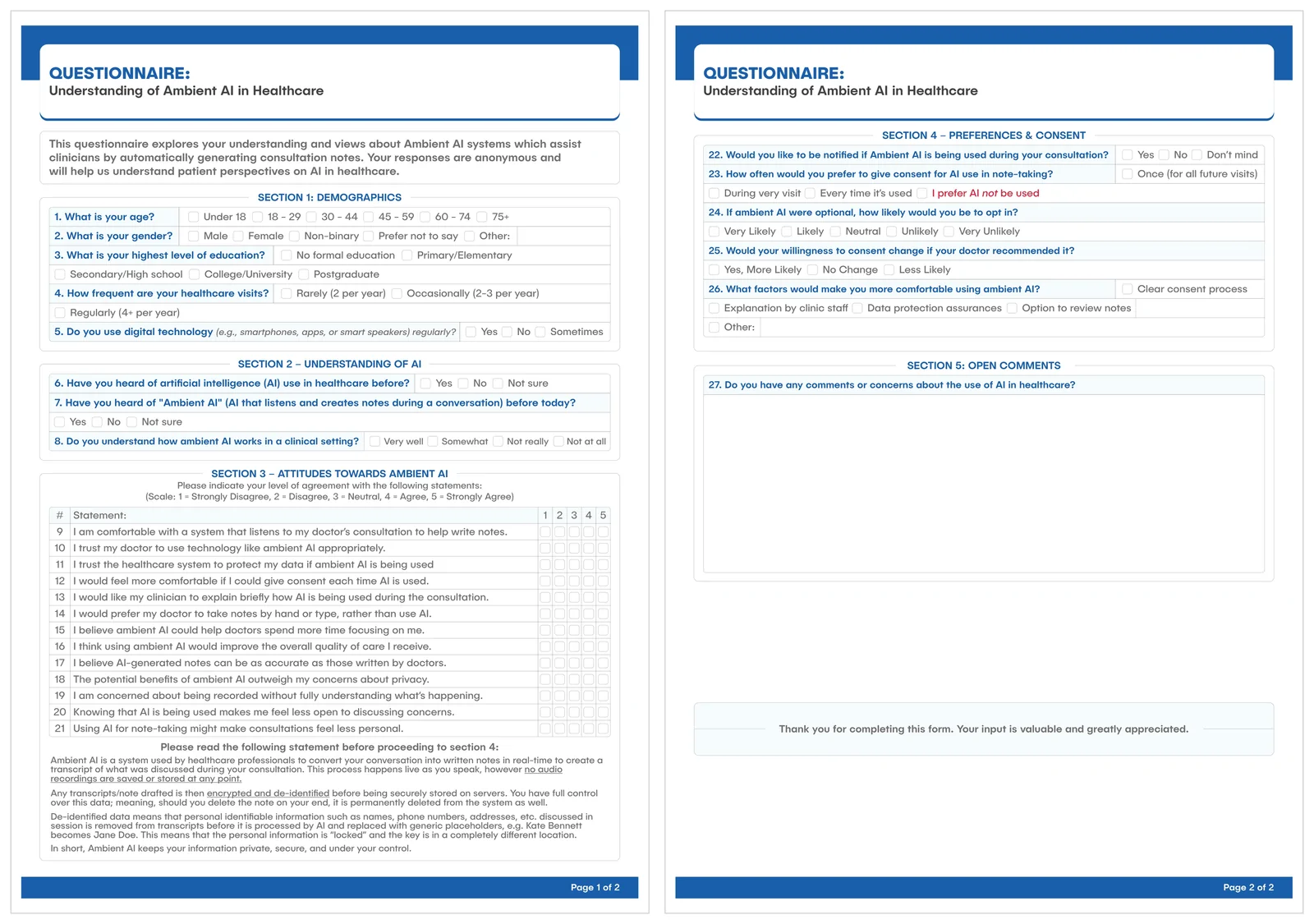
Questionniare

Section 3 consisted of 13 attitudinal statements (Q9-Q21) rated on a five-point Likert scale from strongly disagree to strongly agree. These items explored perceptions relating to comfort, trust, consent, privacy, accuracy and the potential impact of ambient AI on consultation quality. Section 4 examined preferences regarding notification and consent processes, the likelihood of accepting ambient AI use, the influence of clinician recommendations and factors that would increase participants' comfort with the technology. Finally, Section 5 provided an open-ended free-text field for participants to share additional comments and perspectives. 

Data analysis

Categorical variables were summarised using frequencies and percentages. Likert-scale items were summarised using mean and standard deviation. Of the 55 questionnaires returned, one contained demographic responses only and was excluded from item-level analysis; all items from Q8 onward were therefore analysed for 54 respondents. Associations between selected categorical variables were assessed using chi-square tests of independence. Based on response distributions, the following comparisons were examined: prior awareness of ambient AI and willingness to opt in; age group and preference that ambient AI not be used; and digital technology use and prior awareness of ambient AI. A two-sided p-value of <0.05 was considered statistically significant. All data were tabulated and analysed using Microsoft Excel for Mac, version 16.111.1 (Microsoft Corporation, Redmond, WA, USA).

Qualitative free-text comments were analysed using inductive thematic analysis [12], which involved reading the text in full, coding for recurrent concepts, categorising into higher-order themes and then review. Qualitative findings were utilised to contextualise the interpretation of quantitative findings.

## Results

Participant characteristics

All 55 questionnaire responses were analysed. The population was broadly distributed across the adult age spectrum: eight respondents (14.5%) were between 18 and 29 years of age, 11 (20.0%) between 30 and 44 years, 15 (27.3%) between 45 and 59 years, 10 (18.2%) between 60 and 74 years and 11 (20.0%) aged 75 years or older. Twenty-six of the respondents (47.3%) identified as women, 23 respondents (41.8%) identified as men, five respondents (9.1%) identified as non-binary, and one respondent (1.8%) preferred not to say.

The distribution of educational attainment levels was uneven, with the highest number of people having attended a college or university (n=22, 40.0%), followed by secondary or high school education (n=14, 25.5%), postgraduate education (n=13, 23.6%), no formal education (n=4, 7.3%) and primary or elementary education only (n=2, 3.6%). Most participants reported attending healthcare services rarely (n=29, 52.7%), occasionally (n=15, 27.3%) or regularly (n=11, 20.0%). Most respondents (n=45, 81.8%) used digital technology regularly (Table 1). 

**Table 1:** Demographic characteristics of survey respondents (N=55)

Characteristic	Category	n	%
Age group (years)	18-29	8	14.5
	30-44	11	20
	45-59	15	27.3
	60-74	10	18.2
	75+	11	20
Gender	Female	26	47.3
	Male	23	41.8
	Non-binary	5	9.1
	Prefer not to say	1	1.8
Education	No formal education	4	7.3
	Primary/elementary	2	3.6
	Secondary/high school	14	25.5
	College/university	22	40
	Postgraduate	13	23.6
Digital technology use	Regular	45	81.8
	Occasional	6	10.9
	No regular use	4	7.3

Awareness and understanding of AI

Awareness of AI in healthcare was relatively high overall: 40 respondents (72.7%) reported prior awareness, 14 (25.5%) had not heard of it and one (1.8%) was unsure. By contrast, only 24 (43.6%) reported prior awareness of ambient AI specifically, while 22 (40.0%) had not heard of it, and eight (14.5%) were unsure.

Self-reported understanding of ambient AI in clinical settings was limited. Only six respondents (10.9%) reported understanding it "very well", whereas 20 (36.4%) selected "somewhat", 20 (36.4%) selected "not really" and eight (14.5%) selected "not at all". This is summarised in Figure 2.

**Figure 2:**
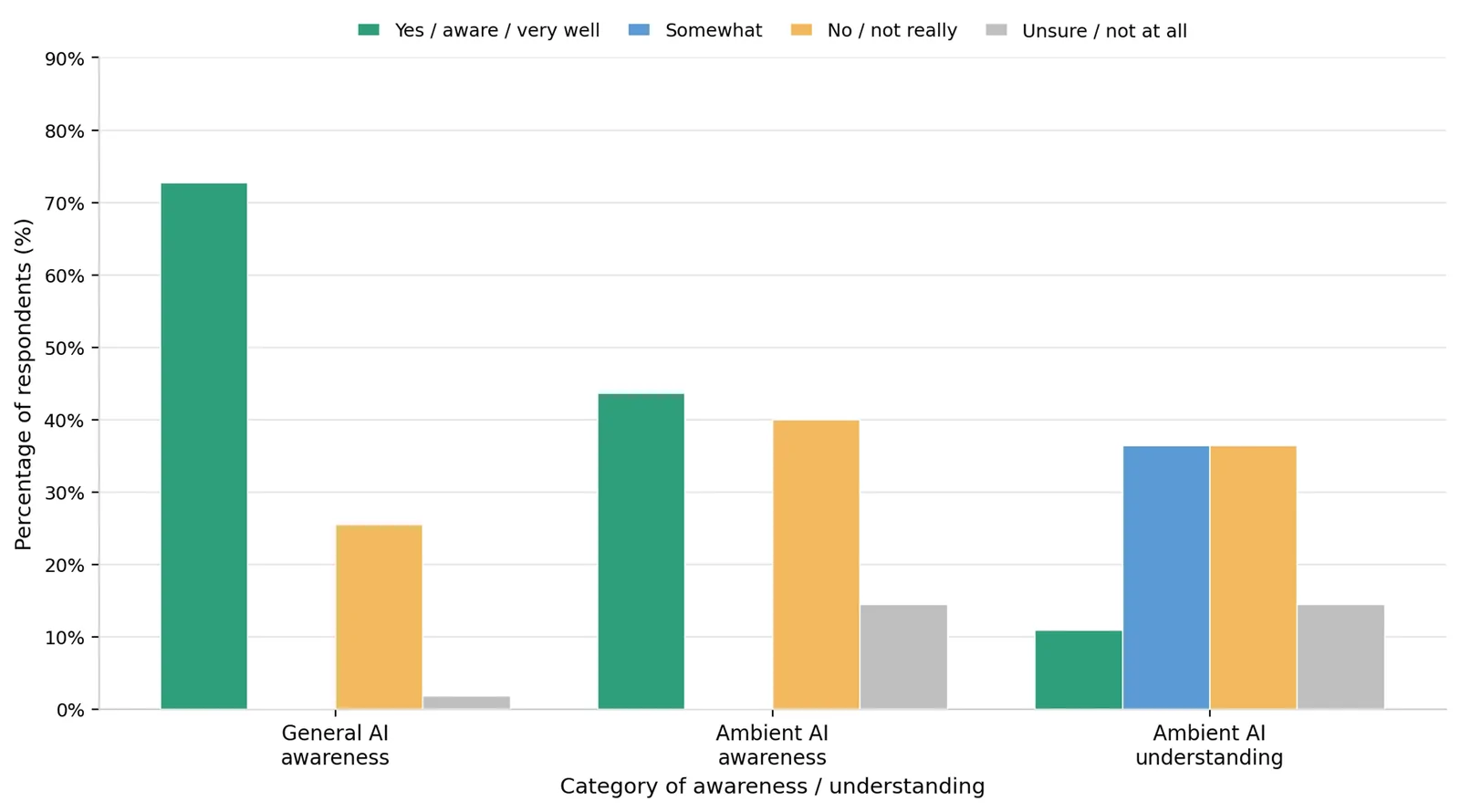
AI awareness levels

Attitudes towards ambient AI

Likert-scale findings showed a pattern of cautious, conditional acceptance of ambient AI seen in Table 2, with most mean scores falling within the neutral-to-agreement range. Participants expressed the strongest support for clinician explanation of AI use during consultations (mean=3.74), indicating that transparency was highly valued. Trust in doctors to use ambient AI appropriately also received a relatively strong endorsement (mean=3.67), alongside moderate comfort with AI systems listening to generate notes (mean=3.59). Acceptance increased when consent was requested for each use (mean=3.52), and respondents moderately agreed that ambient AI could allow clinicians to focus more on patient interaction (mean=3.48).

More reserved attitudes emerged regarding institutional trust, perceived benefits and interpersonal effects. Participants expressed only moderate confidence in healthcare systems protecting personal data (mean=3.35), improving care quality (mean=3.30) and ensuring the accuracy of AI-generated notes (mean=3.24). Privacy concerns remained evident, with many respondents being uncomfortable with being recorded without a full understanding (mean=3.17), and fewer respondents believed benefits outweighed privacy risks. Lower scores for concerns about reduced openness and less personal consultations suggest mixed views rather than outright rejection, showcasing the importance of transparency, consent and maintaining relational aspects of care.

**Table 2:** Mean scores and response distributions for Likert-scale attitude items (Q9-Q21; scale 1-5)

Item	Statement	n	Mean	Agree/SA n (%)	Disagree/SD n (%)
Q9	Comfort with AI listening during consultation	54	3.59	29 (53.7)	11 (20.4)
Q10	Trust in doctor to use ambient AI appropriately	54	3.67	30 (55.6)	8 (14.8)
Q11	Trust in healthcare system to protect data	54	3.35	26 (48.1)	14 (25.9)
Q12	More comfortable if consent obtained each time	54	3.52	30 (55.6)	12 (22.2)
Q13	Clinician should explain how AI is used	54	3.74	32 (59.3)	8 (14.8)
Q14	Prefer manual/typed notes over AI	54	2.89	13 (24.1)	17 (31.5)
Q15	Ambient AI could help doctor focus on patient	52	3.48	26 (50.0)	10 (19.2)
Q16	Ambient AI would improve overall quality of care	54	3.3	23 (42.6)	12 (22.2)
Q17	AI-generated notes as accurate as clinician notes	54	3.24	20 (37.0)	13 (24.1)
Q18	Benefits outweigh privacy concerns	54	3.24	21 (38.9)	13 (24.1)
Q19	Concerned about being recorded without full understanding	54	3.17	24 (44.4)	14 (25.9)
Q20	Would be less open to discussing concerns	54	2.8	16 (29.6)	24 (44.4)
Q21	Consultation would feel less personal	54	2.94	18 (33.3)	20 (37.0)

Notification, consent and opt-in preferences

Half (n=28, 50.9%) said they would prefer to be told if ambient AI was used in their consultation; another 13 (23.6%) said it was a matter of indifference and 13 (23.6%) said they would rather not be told. There was a clear tendency towards active, repeated consent in cases of consent preferences. Most preferred was "consent every time" (n=24, 43.6%), with "consent during every visit" (n=12, 21.8%), "preference that AI not be used at all" (n=12, 21.8%), and "once-for-all-future-visits consent" (n=6, 10.9%) coming next.

If ambient AI were an "opt-in" feature, seven respondents (12.7%) would be very likely to opt in, 19 (34.5%) likely, 15 (27.3%) neutral, 2 (3.6%) unlikely and 11 (20.0%) would be very unlikely to opt in. Therefore, 48.1% of them were likely or very likely to take the option and 24.1% unlikely or very unlikely. The influence of clinician recommendation on consent behaviour was small, with 19 respondents (34.5%) saying they would be more likely to consent if their clinician recommended it, 29 (52.7%) saying they would be no more or less likely to consent and six (10.9%) saying they would be less likely to consent if their clinician recommended it. The preferences are summarised in Table 3.

**Table 3:** Notification, consent and opt-in preferences (N=54)

Domain	Response category	n (N=54)	%
Notification preference (Q22)	Yes - wish to be notified	28	50.9
	Do not mind	13	23.6
	Do not wish to be notified	13	23.6
Consent frequency (Q23)	Every time ambient AI is used	24	43.6
	During every visit	12	21.8
	Prefer AI not be used	12	21.8
	Once for all future visits	6	10.9
Opt-in likelihood (Q24)	Very likely/Likely	26	48.1
	Neutral	15	27.3
	Unlikely/Very unlikely	13	24.1
Effect of clinician recommendation (Q25)	More likely to consent	19	34.5
	No change	29	52.7
	Less likely to consent	6	10.9

There was no statistically significant association between prior awareness of ambient AI and willingness to opt in to its use (χ²(2, N=54)=0.63, p=0.729). There was also no significant association between regular digital technology use and prior awareness of ambient AI (χ²(1, N=54)=1.88, p=0.170).

There was, however, a statistically significant association between age group and the preference that ambient AI not be used (χ²(2, N=54)=7.35, p=0.025). Respondents aged 60 and older were the most likely to express this preference (8/21, 38.1%), compared with 4/18 (22.2%) of those aged 18-44 and 0/15 of those aged 45-59. This pattern was not monotonic across age bands, and the finding should be interpreted with caution given the small cell counts involved.

Factors associated with greater comfort

The list of factors that would make it easier for respondents to use ambient AI was open-ended, allowing them to check off those that would make them feel more comfortable. The most frequently reported factor was data protection assurances (n=23, 41.8%), followed by the ability to review notes (n=17, 30.9%), explanation by clinic staff (n=16, 29.1%) and a clear consent process (n=10, 18.2%). The relative salience of data protection over other elements is consistent with the attitudinal data, which showed that trust in systems trailed behind the trust in clinicians and that confidentiality concerns were prominent.

Qualitative findings

Thematic analysis of free-text responses yielded seven interrelated themes. These are described below in order of prominence.

Theme 1: Conditional Acceptance

One of the most used ideas, rather than unconditional acceptance, was conditional acceptance. There was no uniformity in answers regarding opposition to or endorsement of ambient AI. While there were positive endorsements, there were also carefully qualified affirmations that recognised potential value in specific ways of governance and transparency. This was consistent with the quantitative data, which showed that scores on most attitude items clustered in the moderate range.

*Theme 2: Trust in Clinicians (But Not Necessarily in Systems*)

Ambient AI was readily accepted when presented as a process in control, instead of a process that is autonomous. The final note should be reviewed and endorsed by the doctor as a special note of emphasis noted in the qualitative comments. This was in line with the quantitative difference between the higher mean scores for clinician trust (Q10: mean=3.67) and system-level data protection (Q11: mean=3.35).

Theme 3: Need for Transparency, Explanation and Active Consent

Transparency was a must for acceptability. When asked about the desire to have ambient AI included in their responses, the respondents repeatedly expressed the need to be told when and what it was doing and what it was being used for, and to actively consent and have a meaningful opportunity to agree or decline. This was evident in the highest-scoring quantitative item (Q13), as well as the strong preference for active notification. Ambient AI was not considered a good "background" process. The high level of choice for repeated consent aligned with the notion that the choice of participation in ambient AI recordings should be an active and voluntary one each time. A significant proportion of the minority who were not interested in using AI at all highlights the need for a proper opt-out scheme.

Theme 4: Privacy, Confidentiality and Governance Concerns

Issues of privacy and confidentiality arose as strong concerns to the level of acceptability. The following comments mentioned doubts about the processing, storage and downstream applications of voice data. Concerns related to recording, confidentiality, data handling and the possibility of breach or misuse. Data protection assurances are the top comfort factor (41.8%), indicating substantial data protection concerns. Respondents seemed more worried about governance, control and the ability to determine what happened to their data than about technical security.

Theme 5: Accuracy and Clinical Safety

One theme highly relevant to the clinical context was the potential errors in AI-generated documentation, such as misrecording speech, misinterpretation, incorrect assignment of information or inaccurate transcription, which could pose clinical risks. Several comments referred to the need for strong clinician review and sign-off for acceptability. This theme brings up the topics of patient safety and AI governance.

Theme 6: Perceived Threat to Relational Care/Fear of Depersonalisation

Another less prominent theme was the fear that the personal aspects of the clinical encounter would be lost when dealing with ambient AI. Some of the respondents shared their opinion on care being "human" and how AI could reduce the therapeutic encounter. Comments referred to the importance of preserving one-to-one care and maintaining the relational aspect of clinical encounters. This theme was also reflected in broader concerns about fewer personal consultations and reduced openness. Structured responses were more ambiguous (Q21 mean=2.94; Q20 mean=2.80), suggesting that this is a concern for a minority rather than a majority.

Theme 7: Broader Scepticism Towards AI

One specific subgroup expressed anxieties about ambient AI across healthcare and society in general. One respondent stated concerns about environmental issues associated with the infrastructure needed to power AI, while comments generally referred to AI's "uncontrolled growth" and mentioned its lack of oversight. This means that, for some patients, opposition is based on a deeper concern that extends beyond the level of consultation explanation.

## Discussion

This study was designed to assess patient opinions of ambient AI use in healthcare consultations via a structured survey of 55 patients who had attended an orthopaedic fracture clinic. The results indicate a contingent approach to patients' attitudes, rather than a prescriptive or approving one, characterised by cautious, conditional acceptance rather than simple endorsement or rejection. Theoretically, this position is coherent and consistent with the rest of the technology acceptance literature in the healthcare field, which has consistently identified patient willingness as shaped by perceived benefits, perceived control, trust and risk awareness [8,10,13,14].

The most obvious positive result was that when respondents had a tangible clinical benefit from the use of ambient AI, they were more likely to accept it, including when it could help save clinicians time on administrative tasks and allow for greater attentiveness during consultations (Q15: mean=3.48). This reflects previous findings that patients are more likely to accept health technologies when they believe that they will positively impact their healthcare delivery experience [3,13]. The framing of the benefit for the clinician seems to resonate with the patient's benefit in the ambient AI setting, implying that framing of the benefit of the technology should focus on how it will help keep the clinician present and help improve the quality of interaction.

One of the clearest findings was the difference between trust in clinicians and trust in systems. Respondents were more likely to trust their doctor's use of ambient AI than the healthcare infrastructure that stores and governs data. This was also evident across trust in doctors (Q10: 3.67) and trust in system-level data protection (Q11: 3.35), with mean scores consistently higher for the former, suggesting ambient AI is most acceptable when framed as a clinician-supervised support tool rather than an autonomous or hidden technology. Qualitative responses also highlighted the importance of clinician oversight and review as a precondition for acceptability. The importance of this differential trust is well documented in the patient experience literature [13,14], and this is significant in the context of ambient AI [7]. An institutional commitment to technical security is likely not to be as effective as implementation strategies that emphasise clinician responsibility.

Transparency and consent also emerged as central. Participants strongly favoured explanation by clinicians (Q13: 3.74) and repeated consent over blanket consent (Q12: 3.52). This aligns with broader ethical commentary that ambient listening technologies raise issues beyond simple recording consent, particularly regarding downstream data use, governance and patient understanding [7-9].

A governance perspective on the findings of consent and notification is particularly important. The recommendation of repeated, active consent, agreed to by 43.6% as the correct frequency, is a difficult implementation factor in high-volume clinical settings. Nevertheless, it also embodies the concept of informed, voluntary and specific consent, which has long been recognised as fundamental to the ethical treatment of humans in research and clinical medicine [15]. The General Data Protection Regulation (GDPR) and the applicable health data frameworks in the UK also endorse the idea of opt-in consent for the use of sensitive personal information rather than opt-out [16]. The current results suggest that patients hold this expectation and could perceive implied or one-time consent as too low. This expectation among patients is echoed by clinicians. In a survey of 300 dermatologists across 13 countries, almost 60% felt that patient consent should be mandatory when AI tools are used, which the authors took as evidence of broad professional support for patient autonomy. Notably, this view was more common among dermatologists who had never used AI themselves, suggesting greater caution among non-users. That both patients in our study and clinicians elsewhere favour explicit consent points to a shared expectation rather than a purely regulatory one, and supports building recurring consent into ambient AI workflows [17].

Another interesting thing to note is the low magnitude of the influence of the clinician's recommendation on consent behaviour, as 34.5% of the participants said a recommendation would make them more likely to agree, but the majority (52.7%) reported that nothing would change. This could be a divide between what patients are told to do and what the administration tells them to do, and they want to keep their own data and do with it as they please [10]. Services should not take it upon themselves to make ambient AI accepted just because the clinical community endorses it.

Concerns about misinterpretation, misattribution and resulting clinical error were supported by lower mean scores on the equivalence of AI-generated and clinician-authored notes (Q17: mean=3.24). This places ambient AI in the line of patient safety. Previous studies on clinical AI note that the outputs of language models may suffer from transcription errors and incorrect context [18,19], and these results indicate that patients are aware of this risk. There should be clear, patient-oriented communication about the review/validation process in implementation governance, as well as audit systems to identify and rectify systematic documentation errors.

The qualitative findings also show that patients are attentive to more than efficiency. Concerns about privacy, confidentiality, note accuracy and depersonalisation suggest that ambient AI may affect the perceived quality of the consultation and its administrative efficiency. The significant association between older age and preference for non-use further suggests that implementation may need to be sensitive to age-related differences in comfort or trust.

Notably, prior awareness of ambient AI was not significantly associated with willingness to opt in. This suggests that familiarity alone may be insufficient to improve acceptability if broader concerns around trust, communication and governance remain unaddressed.

The benefits and drawbacks of ambient AI vary considerably across clinical specialities, with direct implications for the generalisability of single-setting studies such as this one. Specialities characterised by highly technical language, rapid task-switching or a heavy procedural focus - such as surgery, emergency medicine, and some subspecialty clinics - often see limited benefit, as error rates and review burden may outweigh the time saved. High-volume, conversational visit types tend to perform better than procedural or highly technical encounters. With the development of telemedicine, ambient AI could be utilised in virtual healthcare settings [20].

Strengths and limitations

A strength of this study is its mixed-methods design, which allowed both numerical characterisation of attitudes and deeper exploration of patient concerns. The combination of structured attitudinal items and thematic analysis provided a fuller account of patient acceptability.

There are several caveats. Only descriptive statistical analysis was performed due to the relatively small sample size of 55; this limits statistical precision and generalisability, and the results should be treated as hypothesis-generating. Recruitment from an orthopaedic fracture clinic may not be representative of patients in other clinical settings (e.g. primary care, mental health services). The study used convenience sampling, which may have introduced selection bias, as individuals who refused to participate may have had different perspectives. Self-reported knowledge of ambient AI may not reflect actual knowledge, which prior experiences, assumptions or varying interpretations of ambient AI may influence.

The findings would be greatly expanded in future research with larger multi-site samples, validated attitudinal scales and by applying purposive qualitative sampling methods.

## Conclusions

Our findings indicate that the ethical and effective implementation of ambient AI in health encounters is not possible without users' awareness. Furthermore, the fact that some respondents opposed the use of AI, regardless of the circumstances, emphasises the need to keep patient choice. There needs to be meaningful opportunities, therefore, to refuse to participate, which is a required safeguard. This research has several implications for healthcare organisations interested in deploying ambient AI systems. Plans for implementing technology should utilise communication strategies to explain the purpose, operation and governance of the technology, using language accessible to a range of patient populations. Consent processes should facilitate re-consent for multiple and specific consents, not just one-off authorisations. The clinical governance structures should also have clear processes on how clinicians will check, validate and sign AI-generated records prior to finalising the documentation. Patient experience, documentation, opt-out trends and the effectiveness of the consultation should be continuously monitored to track over time.

The findings and recommendations of this study highlight the need for transparency, patient autonomy and the clinician's long-term responsibility as key elements in the implementation of ambient AI. It is also important that patients are given clear information about their rights to refuse to take part in any procedure without this affecting the quality of the care they receive. Institutions are encouraged to make available information that is easy to access regarding voice recordings and associated data, including how they are processed, stored, accessed and deleted. Opportunities to access (and review) generated summaries may help increase confidence and, where possible, reinforce perceptions of accuracy. Ambient AI has the potential to deliver real value by streamlining administrative burdens and giving clinicians more time to focus on patient interaction during clinical consultations. The equitable achievement of these benefits, however, will hinge on the importance of design and implementation factors such as patient acceptability, rather than being an afterthought.
